# Exogenous Oleic Acid and Palmitic Acid Improve Boar Sperm Motility via Enhancing Mitochondrial Β-Oxidation for ATP Generation

**DOI:** 10.3390/ani10040591

**Published:** 2020-03-31

**Authors:** Zhendong Zhu, Rongnan Li, Chengwen Feng, Ruifang Liu, Yi Zheng, S. A. Masudul Hoque, De Wu, Hongzhao Lu, Tao Zhang, Wenxian Zeng

**Affiliations:** 1 Key Laboratory of Animal Genetics, Breeding and Reproduction of Shaanxi Province, College of Animal Science and Technology, Northwest A&F University, Yangling 712100, China; zhendongzhu@nwafu.edu.cn (Z.Z.); lirongnan2016@nwafu.edu.cn (R.L.); fengchengwen585@163.com (C.F.); liuruifang79@163.com (R.L.); y.zheng@nwafu.edu.cn (Y.Z.); 2 Department of Animal Breeding and Genetics, Bangabandhu Sheikh Mujibur Rahman Agricultural University, Gazipur 1706, Bangladesh; mhoqueabg@bsmrau.edu.bd; 3 Key Laboratory for Animal Disease Resistance Nutrition of the Ministry of Education of China, Institute of Animal Nutrition, Sichuan Agricultural University, Chengdu 611130, China; wude@sicau.edu.cn; 4 College of Biological Science and Engineering, Shaanxi University of Technology, Hanzhong 723001, China; zl780823@126.com

**Keywords:** motility, boar, fatty acid, metabolism, β-oxidation

## Abstract

**Simple Summary:**

Sperm requires ATP production for maintaining motility. In boar sperm, it is not clear whether the mitochondrial β-oxidation pathway for ATP generation is active or not. We found that boar sperm could utilize oleic acid and palmitic acid during the liquid storage. Addition of oleic acid and palmitic acid to extender improved the sperm quality. Using the incubation model, we found that boar sperm utilized oleic acid and palmitic acid as the energy substrates for ATP generation via mitochondrial β-oxidation pathway. We suggest that addition of fatty acids to the extender would be beneficial to improve boar sperm quality.

**Abstract:**

It takes several hours for mammalian sperm to migrate from the ejaculation or insemination site to the fertilization site in the female reproductive tract in which glucose, amino acids, and fatty acids are regarded as the primary substrates for ATP generation. The present study was designed to investigate whether oleic acid and palmitic acid were beneficial to boar sperm in vitro; and if yes, to elucidate the mechanism that regulates sperm motility. Therefore, the levels of oleic acid and palmitic acid, motility, membrane integrity, acrosome integrity, and apoptosis of sperm were evaluated. Moreover, the enzymes involved in mitochondrial β-oxidation (CPT1: carnitine palmitoyltransferase 1; ACADVL: long-chain acyl-coenzyme A dehydrogenase) were detected with immunofluorescence and Western blotting. Consequently, the ATP content and the activities of CPT1, ACADVL, malate dehydrogenase (MDH), and succinate dehydrogenase (SDH) were also measured. We observed that CPT1 and ACADVL were expressed in boar sperm and localized in the midpiece. The levels of oleic acid and palmitic acid were decreased during storage at 17 °C. The addition of oleic acid and palmitic acid significantly increased sperm motility, progressive motility, straight-line velocity (VSL), membrane integrity, and acrosome integrity with a simultaneous decrease in sperm apoptosis after seven days during storage. When sperm were incubated with oleic acid and palmitic acid at 37 °C for 3 h, the activities of CPT1 and ACADVL, the ATP level, the mitochondrial membrane potential, the activities of MDH and SDH, as well as sperm motility patterns were significantly increased compared to the control (*p* < 0.05). Moreover, the addition of etomoxir to the diluted medium in the presence of either oleic acid or palmitic acid and the positive effects of oleic acid and palmitic acid were counteracted. Together, these data suggest that boar sperm might utilize oleic acid and palmitic acid as energy substrates for ATP production via β-oxidation. The addition of these acids could improve sperm quality.

## 1. Introduction 

In mammals, sperm motility is essential for sperm migration from the ejaculation sites to the oviduct for fertilization in vivo [1], where the motility is governed by ATP generation [2]. The mitochondrial β-oxidation is one of the metabolic pathways for ATP production in mammalian cells [3,4]. Cells generate ATP via different metabolic pathways depending on the composition of metabolic substrates in the microenvironment [5,6]. Amino acids and pyruvate are rich in uterus fluids, which sperm utilize as energy substrates through the mitochondrial oxidative phosphorylation pathway [7,8,9]. On the contrary, glycolysis is the primary pathway in the oviduct site due to the high glucose content in oviduct fluids [10,11]. In addition, Zhu et al. [2] reported that sperm could use different energy substrates for ATP production via different metabolic pathways for maintaining sperm motility. Therefore, supplementation of exogenous energy substrates to the extender may enhance sperm survival during the liquid storage.

Previous studies reported that a diet supplemented with fish oil that was a rich source of long-chain fatty acids significantly improved sperm motility and semen production in boar [12], human [13], and ram [14]. Oleic acid and palmitic acid are two of the prominent saturated fatty acids in sperm [15,16]. Amaral et al. [17] reported that a large proportion of the metabolic proteome (24%) comprised enzymes that were involved in lipid metabolism, including enzymes for β-oxidation in human sperm. Baker et al. [18] also found that delta (3,5)-delta (2,4)-dienoyl-CoA isomerase (ECH1), an enzyme involved in β-oxidation, existed in human sperm. Interestingly, inhibition of β-oxidation using etomoxir led to a decrease of sperm motility, suggesting that the mitochondrial β-oxidation metabolic pathway is an active regulator for sperm motility in humans [17]. Moreover, it was reported that supplementation of palmitic acid or oleic acid to the extender improved sperm quality in bulls [19]. Hossain et al. [20] also found that exposure of boar sperm to unsaturated free fatty acids enhanced motility and viability during in vitro incubation. However, the mechanism by which palmitic acid or oleic acid protects sperm quality is unknown. Since the fatty acids are easily incorporated into the cell membrane and act as substrates for ATP production via mitochondrial β-oxidation [21,22], we hypothesized that palmitic acid and oleic acid might contribute to ATP production via mitochondrial β-oxidation to protect sperm against energy stress in vitro. Therefore, the present study was performed to investigate whether oleic acid and palmitic acid were beneficial to boar sperm during liquid storage, and if yes, then to elucidate the underlying mechanism.

## 2. Materials and Methods

### 2.1. Reagents and Media

All reagents were purchased from Sigma Chemical (Shanghai, China), unless otherwise specified. 

### 2.2. Semen Collection and Processing

All animals and experimental procedures were approved by the Northwest A&F University Institutional Animal Care and Use Committee. Five mature and reproductively good duroc boars (aged 15–28 months) were used in this study. The boars were housed individually, maintained under natural daylight, fed basal diets, and provided free access to water. The sperm-rich ejaculate fraction was collected weekly from each boar using the gloved-hand method. Ejaculated semen was placed in flasks with isolated containers at 37 °C and processed in the laboratory within 30 min after collection. Only ejaculates containing more than 80% motile sperm (evaluated by phase-contrast microscopy) were used in this study. Fresh semen was diluted with the modified Modena solution according to Zhu et al. [2]. The modified Modena solution was composed of 30.6 mM glucose, 122.4 mM lactose, 26.7 mM trisodium citrate, 11.9 mM sodium hydrogen carbonate, 15.1 mM citric acid, 6.3 mM EDTA-2Na, 46.6 mM Tris, 1000 IU/mL penicillin G potassium, and 1 mg/mL amikamycin. The final concentration of the diluted sperm was 3.0 × 10^7^sperm/mL. The diluted semen was split into 80 mL semen doses and stored at 17 °C in a cool incubator (BC-43KT1, Hisense Co., Qingdao, China) before evaluation.

### 2.3. Oleic Acid and Palmitic Acid Analysis

Fatty acid analyses in sperm were performed using gas chromatography [23] in samples during liquid storage at 17 °C in a cool incubator. According to Martinez-Soto et al. [24], 10^8^ spermatozoa were resuspended with 300 μL PBS and transferred into glass tubes for direct transesterification. Each sample was added with 2 mL methanol-benzene (4:1, v/v) that contained an internal standard (heptadecanoic acid, C17:0) and 0.01% butylhydroxytoluene as an antioxidant. Then, two hundred microliters of acetyl chloride were slowly added to the samples with vortexing at low speed. Samples were then heated for 2 h at 80 °C in a heating block and shaken continuously. Five milliliters of 6% potassium carbonate (w/v) were then added to the samples after the tubes had been cooled to room temperature. The samples were centrifuged at 5000× *g* for 10 min at 15 °C after being vortexed for 30 s. The upper benzene phase (containing fatty acid methyl esters (FAMEs)) was transferred to gas chromatography vials and stored at 4 °C until chromatograph evaluation.

The analysis was performed on a Varian CP-3900 Gas Chromatograph (Varian Inc., Palo Alto, CA, USA) equipped with a flame ionization detector, using a capillary column Model CP9205-VF-WAXms (Agilent Technologies Espana S.L., Madrid, Spain). The chromatograms with peak retention times and areas were produced on the recording integrator and were electronically transferred to the computer for analysis, storage, and report generation. Peak naming and column performance were achieved according to the calibration standard mix. Oleic acid and palmitic acid were identified by the order of elution and compared with known commercially prepared fatty acid standards GLC 566-C (Nu-Chek Prep Inc., Elysian, MN, USA). 

### 2.4. Assessment of Sperm Motility

According to Zhu et al. [2], the computer-assisted sperm motility analysis (CASA) system (HT CASA-Ceros II; Hamilton Thome, MA, United States) was used to evaluate sperm motility. Briefly, a 5 μL aliquot of semen was placed on the analyzer’s Makler chamber maintained at 37 °C during the analysis. Three fields were selected for computer-assisted analysis.

### 2.5. Sperm Membrane Integrity and Acrosome Integrity

The LIVE/DEAD Sperm Viability Kit (Leiden the Netherlands, L7011) and fluorescein isothiocyanate-peanut agglutinin (FITC-PNA) were used to evaluate sperm membrane integrity and acrosome integrity, respectively, according to Zhu et al. [25,26]. The stained sperm was monitored and photographed by an epifluorescence microscope (Nikon 80i; Tokyo, Japan) with a set of filters (200×) with 535 nm excitation and 617 nm emission for red fluorescence and 488 nm excitation and 525 nm emission for green fluorescence.

### 2.6. Mitochondrial Membrane Potentials

The JC-1 (5,5′,6,6′-tetrachloro-1,1′,3,3′-tetraethyl benzimidazole-carbocyanine iodide) Mitochondrial Membrane Potential Detection Kit (Beyotime Institute of Biotechnology, China) was used to analyze sperm mitochondrial membrane potentials according to our previous study [2]. Monomer and aggregates were the two types of JC-1 in stained mitochondrial plasma. Sperm with low mitochondrial membrane potential showed the monomer that emitted green fluorescence, while sperm with high mitochondrial membrane potential emitted red fluorescence. A monochromator microplate reader (Safire II, Tecan, Switzerland) was used to analyze the fluorescence intensity of both mitochondrial JC-1 monomers (λex 514 nm, λem 529 nm) and aggregates (λex 585 nm, λem 590 nm). The Δψm of sperm in each treatment group was calculated as the fluorescence ratio of red (aggregates) to green (monomer). Analyses were performed in triplicate (n = 3).

### 2.7. Annexin V-FITC/PI Assay

The Annexin V-FITC/PI apoptosis detection kit (Sigma-Aldrich, St. Louis, MO, USA) was used to assess sperm apoptosis according to the manufacturer’s instruction. The sperm sample was incubated with Annexin V-FITC/PI working solution at a concentration of 1 × 10^6^ sperm/mL at room temperature for 10 min in the dark. The stained sperm was monitored and photographed with an epifluorescence microscope (80i; Nikon). Analyses were performed in triplicate (n = 3).

### 2.8. Assessment of ATP level

The ATP Assay Kit (Beyotime Institute of Biotechnology, China) was used to measure the sperm ATP level. According to the previous study, the sample was added to the luciferin/luciferase reagent in the 96 well plate. The luminescence at integration × 1000 ms was read using an Ascent Luminoskan luminometer (Thermo Scientific, Palm Beach, FL) with phosphate-buffered saline as a blank for each experiment. A standard curve was constructed using standards containing 0.01, 0.03, 0.1, 0.3, 1, 3, and 10 μM ATP. Analyses were performed in triplicate (n = 3).

### 2.9. Assessment of Sperm MDH and SDH Activities 

The MDH and SDH activities were measured using a Malate Dehydrogenase assay kit and a Succinate Dehydrogenase assay kit (Nanjing Jiancheng Bioengineering Institute), respectively. According to Zhu et al. [27], sperm samples were lysed ultrasonically (20 kHz, 750 W, operating at 40% power, 5 cycles of 3 s on and 5 s off) and centrifuged at 2000× *g* for 10 min at 4 °C. The supernatants were added to a 96 well plate for the analysis of MDH and SDH activities using a microplate reader at 340 nm and 600 nm, respectively. MDH and SDH activities were expressed as mU per mg protein. Protein concentrations were measured with a BCA assay kit. Analyses were performed in triplicate (n = 3).

### 2.10. Immunolocalization of CPT1 and ACADVL in Boar Sperm by Immunofluorescence

Sperm samples were washed with PBS, fixed with 4% paraformaldehyde for 10 min, spread onto the poly-L-lysine slides, and air dried at room temperature. The samples were permeabilized with 0.5% Triton X-100 in PBS for 10 min. Nonspecific binding was blocked with PBS in the presence of 10% BSA (Sigma-Aldrich) for 30 min at room temperature. Samples were then incubated with anti-CPT1(sc-514555; 1:25) and anti-ACADVL (sc-271225; 1:50) overnight at 4 °C. The samples were rinsed with PBS 3 times and then incubated with biotinylated goat anti-mouse FITC-IgG (1:200) the next day. Sperm were washed and counterstained with DAPI (CWBIO). Fluorescent images were captured with fluorescence microscopy (80i, Nikon, Japan). Negative control immunostaining was also performed at the same time without the primary antibody.

### 2.11. Assessment of Sperm CPT1 and ACADVL Activities

The CPT1 and ACADVL activities were measured using a carnitine palmitoyltransferase 1 assay kit (Nanjing Jiancheng Bioengineering Institute) and a long-chain acyl-coenzyme A dehydrogenase assay kit (Oulu Biotechnology), respectively. Sperm samples were lysed ultrasonically (20 kHz, 750 W, operating at 40% power, 5 cycles of 3 s on and 5 s off) and centrifuged at 2000× *g* for 10 min at 4 °C. The supernatants were used to analyze the activities of CPT1 and ACADVL according to the manufacture’s protocols. Analyses were performed in triplicate (n = 3).

### 2.12. Western Blotting

Total proteins were extracted from sperm under different treatments with lysis buffer. The protein concentration was measured with a BCA assay kit (TaKaRa, Dalian, China). The proteins were then separated by 12.5% SDS-PAGE and transferred to a PVDF membrane. The membrane was blocked with 5% BSA for 2 h and then incubated with anti-cleave caspase3 (CST, 1:1,000), anti-cleave caspase 9 (CST, 1:1,000), anti-p53 (CST, 1:1,000), anti-Parp-1 (CST, 1:1,000), anti-CPT1 (Santa Cruz,1:1,000), anti-ACADVL (Santa Cruz,1:1,000), and anti-α-tubulin (Santa Cruz,1:1,000) at 4 °C overnight. HRP conjugated goat anti-rabbit and goat anti-mouse antibodies were used as the secondary antibody, respectively (1:2000 final dilution). The reagent for enhanced chemiluminescence (Bio-Rad) was used for detection and developed by X-ray film (Champchemi Top610, China).

### 2.13. Experiment Design

Experiment 1 aimed to evaluate whether the exogenous oleic acid and palmitic acid were beneficial to boar sperm diluted with modified Modena solution during the liquid storage at 17 °C for 7 days. Firstly, the change of oleic acid and palmitic acid levels was measured at 0, 1, 3, 5, and 7 day points of the storage. Secondly, sperm were exposed to different concentrations of oleic acid (OA: 0, 50, 100, 150, 200 μM) and palmitic acid (PA: 0, 25, 50, 75, 100 μM) during liquid storage. Sperm motility, membrane integrity, acrosome integrity, and apoptosis were also assessed after 7 days of storage.

Experiment 2 was to investigate whether sperm metabolized oleic acid and palmitic acid via β-oxidation to generate ATP to maintain sperm quality. Firstly, the expression and localization of the CPT1 and ACADVL enzymes in boar sperm were identified by Western blotting and immunofluorescence, respectively. Secondly, the fresh semen was diluted with modified Modena solution at a concentration of 3.0 × 10^7^ and incubated with different concentrations of oleic acid (0, 50, 100, 150, 200 μM) and palmitic acid (0, 25, 50, 75, 100 μM) at 37 °C for 3 h. The CPT1 and ACADVL activities, mitochondrial membrane potential, ATP content, MDH and SDH activities, as well as the sperm motility patterns were analyzed after 3 h of incubation.

Experiment 3 was to confirm further that sperm utilize oleic acid and palmitic acid using the CPT1 selective inhibitor, etomoxir, to block sperm β-oxidation. Sperm were incubated with different concentrations (0, 1, 10, 100, 1000 μM) of etomoxir for 3 h, and the percentage of dead sperm, progressive motility, and straight-line velocity (VSL) and CPT1 activity were evaluated after 3 h of incubation. Secondly, one-hundred micromolar etomoxir was used for incubation with sperm in the following experiment: (1) a group incubated with 100 μM OA; (2) a group incubated with 100 μM OA and 100 μM etomoxir; (3) a group incubated with 75 μM PA; and (4) a group incubated with 75 μM PA and 100 μM etomoxir. 

### 2.14. Statistical Analysis

All data were tested for normality and variance of homogeneity prior to statistical analysis. If necessary, the data were transformed with arc-sin square root transformation. Data were analyzed by one-way ANOVA and analyzed with multiple comparisons with the Tukey test using SPSS Version 17.0 for Windows (SPSS Inc., Chicago, IL). All the values are presented as the mean ± standard deviation (SD). Treatments were considered statistically different from one another at *p* < 0.05.

## 3. Results

### 3.1. Levels of OA and PA in Boar Sperm During Storage

**Figure 1:**
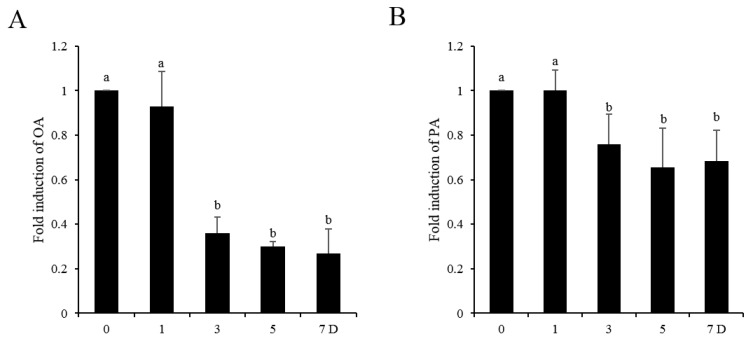
The levels of OA (**A**) and PA (**B**) in sperm were decreased during the liquid storage at 17 °C. Values are specified as the mean ± standard deviation (SD). Columns with different lowercase letters differ significantly (*p* < 0.05). OA: oleic acid. PA: palmitic acid.

As shown in Figure 1A, the OA level in sperm was not changed in the first day, but significantly decreased at 3 days, 5 days, and 7 days of storage compared to that of fresh sperm. The level of PA was also not changed after one day of storage, but significantly decreased after three days of storage (Figure 1B). 

### 3.2. Addition of OA and PA Affected Boar Sperm Motility Patterns, Membrane Integrity, and Acrosome Reaction during Storage

**Figure 2:**
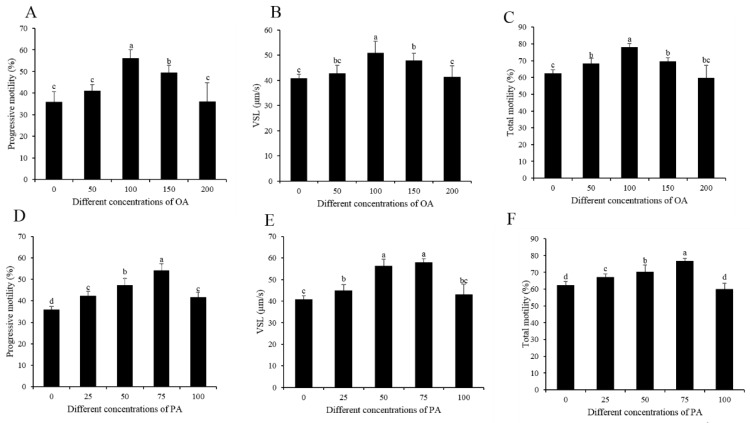
(**A**–**D**) Dynamic changes in the sperm parameters by the addition of OA and PA to the diluted medium after seven days of preservation: (**A**,**D**) progressive motility, (**B**,**E**) VSL, and (**C**,**F**) total motility. Values are specified as the mean ± standard deviation (SD). Columns with different lowercase letters differ significantly (*p* < 0.05). VSL: straight-line velocity.

As the oleic acid and palmitic acid levels were decreased during liquid storage, different concentrations of OA (0, 50, 100, 150, 200 μM) and PA (0, 25, 50, 75, 100 μM) were added to the extender to elucidate whether they improved the sperm quality or not. It was observed that the addition of 100 and 150 μM OA significantly increased the total motility, progressive motility, and VSL of sperm, whereas the 50 and 200 μM OA treatments were similar to the control (Figure 2A–C). Interestingly, the 100 μM OA treatment showed the highest values of the sperm motility patterns among all the OA treatments (Figure 2A–C). The addition of PA at 25, 50, and 75 μM concentration also significantly improved sperm total motility, progressive motility, and VSL upon storage (Figure 2D–F); however, these motility patterns were decreased at 100 μM level (Figure 2D–F). The results of sperm motility patterns showed that 75 μM was the most effective concentration for palmitic acid improving sperm quality (Figure 2D–F).

**Figure 3:**
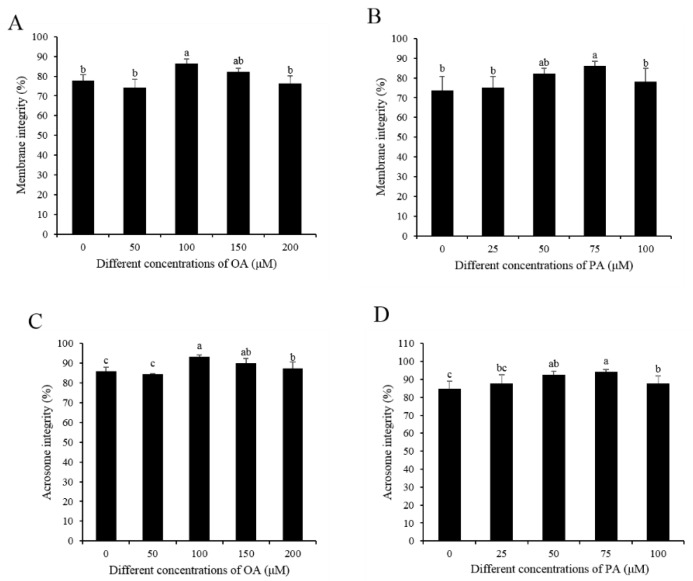
Effects of OA and PA on sperm membrane integrity and acrosome integrity after seven days of preservation: (**A**,**B**) membrane integrity; (**C**,**D**) acrosome integrity. Values are specified as the mean ± standard deviation (SD). Columns with different lowercase letters differ significantly (*p* < 0.05).

In terms of sperm membrane integrity, the addition of both OA (100 μM) and PA (75 μM) to the extender significantly increased sperm membrane integrity after seven days of storage (Figure 3A,B). Moreover, the percentage of sperm with intact acrosome was increased by the addition of different concentrations of OA (100 and 150 μM) and PA (50 and 75 μM) to the extender (Figure 3C,D). 

### 3.3. Effects of OA and PA on Boar Sperm Apoptosis during the Liquid Storage

**Figure 4:**
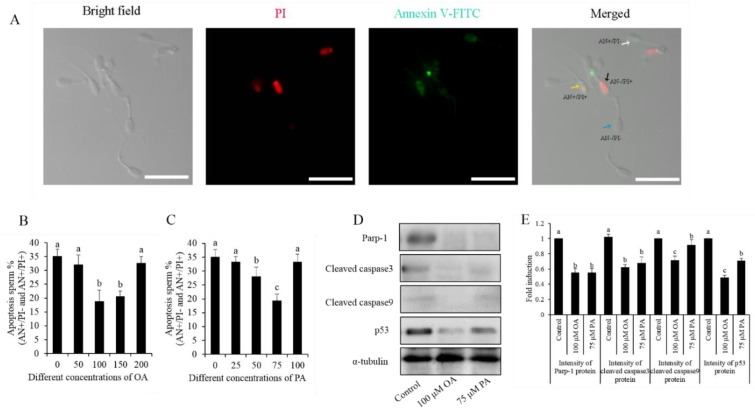
(**A**) Photomicrographs of the boar sperm stained with the Annexin V-FITC/PI assay kit: live sperm (AN-/PI-; blue arrow); early apoptotic sperm (AN+/PI-; white arrow); late apoptotic sperm (AN+/PI+; yellow arrow); and non-viable necrotic sperm (AN-/PI+; black arrow). Effects of different concentrations of OA (**B**) and PA (**C**) on boar sperm apoptosis. The Western blotting image shows the expression of apoptosis proteins in boar sperm after being treated with OA and PA (**D**). (**E**) Quantitative expression of the Parp-1, cleaved caspase-3, cleaved caspase-9, and p53 over α-tubulin generated from Western blotting (**D**). Data are the mean ± standard deviation (SD). Columns with different lowercase letters differ significantly (*p* < 0.05). Bars = 30 μm.

As shown in Figure 4A, the sperm stained with Annexin V-FITC/PI was classified into four groups: live sperm (AN-/PI-; blue arrow); early apoptotic sperm (AN+/PI-; white arrow); late apoptotic sperm (AN+/PI+; yellow arrow); and non-viable necrotic sperm (AN-/PI+; black arrow). It was observed that the addition of either oleic acid or palmitic acid significantly decreased the apoptosis of sperm (AN+/PI- and AN+/PI+ groups), where 100 μM OA and 75 μM PA treatments showed the lowest percentage of sperm with apoptosis (Figure 4B,C). In the results of Western blotting detection, it was observed that the expression of apoptotic factor proteins (Parp-1, cleaved-caspase 3, cleaved-caspase 9, and p53) was also significantly decreased by either the 100 μM OA or 75 μM PA treatment, compared to the control. (Figure 4D,E).

### 3.4. Localization and Expression of the Enzymes, CPT1, and ACADVL, Involved in β-oxidation in Boar Sperm

**Figure 5:**
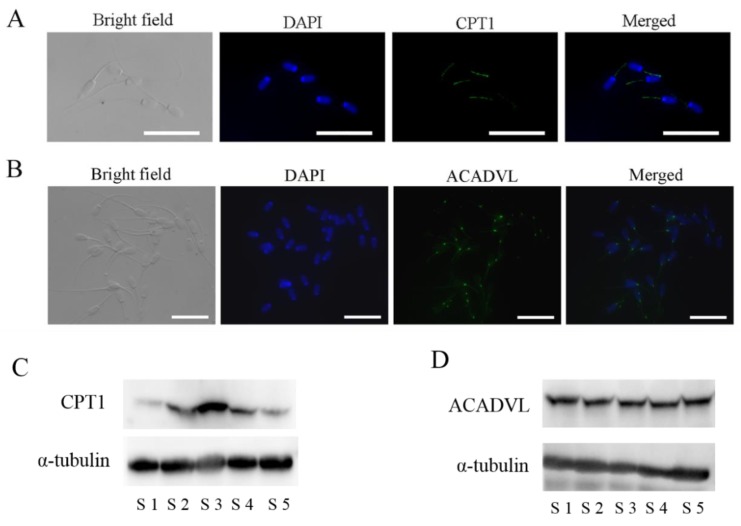
Detection and immunofluorescent localization of CPT1 (**A**) and ACADVL (**B**) in boar sperm. Western blotting analysis of CPT1 (**C**) and ACADVL (**D**) in fresh boar sperm. Bars = 30 μm. CPT1: carnitine palmitoyltransferase 1. ACADVL: long-chain acyl-coenzyme A dehydrogenase. S1-S5: five different boar sperm samples.

To test the hypothesis that boar sperm may utilize oleic acid and palmitic acid for ATP production via β-oxidation to maintain sperm motility, firstly, the CPT1 and ACADVL enzymes that were involved in β-oxidation were detected in boar sperm using Western blotting and immunofluorescence. CPT1 is an enzyme necessary for the transport of long-chain fatty acids into the mitochondrial matrix and hence for mitochondrial β-oxidation. The CPT1 protein was expressed in fresh boar sperm and mainly localized at the midpiece, where the mitochondria are distributed (Figure 5A,C). Moreover, ACADVL, an enzyme involved in the first step of fatty acid β-oxidation, was also expressed and localized in the midpiece, especially in the junction between sperm head and midpiece (Figure 5B,D). 

### 3.5. Effects of OA and PA on CPT1 and ACADVL Activities, Mitochondrial Activity, MDH and SDH activities, ATP Levels, as well as Sperm Linear Motility during the 3 h of Incubation

**Figure 6:**
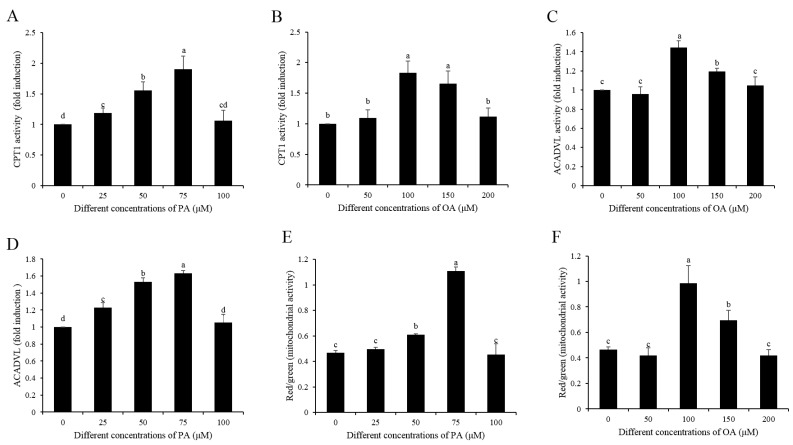
(**A**–**D**) Dynamic changes of CPT1 activity, ACADVL activity, and mitochondrial activity by the addition of OA and PA to the diluted medium at the 3h point of incubation: (**A**,**B**) CPT1 activity, (**C**,**D**) ACADVL activity, and (**E**,**F**) mitochondrial activity. Data are the mean ± standard deviation (SD). Columns with different lowercase letters differ significantly (*p* < 0.05).

**Figure 7:**
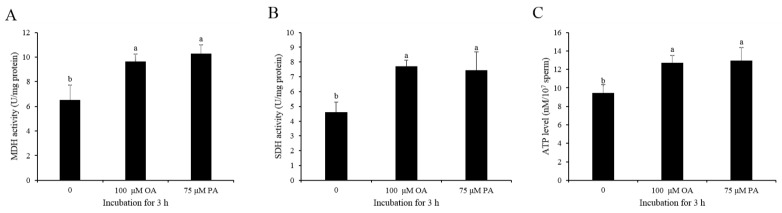
Addition of OA and PA increased sperm MDH activity (**A**), SDH activity (**B**), and ATP level (**C**) after 3h of incubation. Data are the mean ± standard deviation (SD). Columns with different lowercase letters differ significantly (*p* < 0.05). MDH: malate dehydrogenase. SDH: succinate dehydrogenase.

The addition of either OA (100 and 150 μM) or PA (25, 50, and 75 μM) to the diluted medium significantly increased the activities of CPT1 and ACADVL after 3 h of incubation (Figure 6A–D). Interestingly, when compared to the control, the addition of either oleic acid or palmitic acid significantly increased sperm mitochondrial activity (Figure 6E,F). MDH and SDH are the two enzymes of the TCA cycle; high activities of MDH and SDH indicate sperm with high TCA cycle activity for ATP generation status. In the present study, when sperm incubated with either OA or PA, the activities of MDH and SDH were significantly increased (Figure 7A,B). Moreover, the sperm ATP level was also significantly increased when sperm was treated with OA and PA during the 3 h of incubation (Figure 7C).

**Figure 8:**
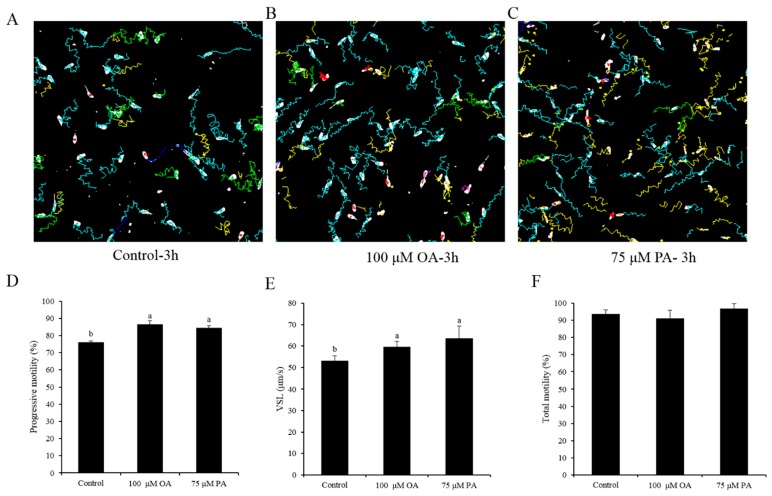
(**A**–**C**) Computer-assisted sperm motility analysis (CASA) derived changes in sperm motility track by the addition of OA and PA to the diluted medium. (**D**–**F**) Dynamic changes in the sperm parameters: (**D**) progressive motility, (**E**) VSL, and (**F**) total motility. Data are the mean ± standard deviation (SD). Columns with different lowercase letters differ significantly (*p* < 0.05).

As shown in Figure 8A–C, the motility tracks generated by CASA revealed that sperm linear motility was also improved by the addition of oleic acid and palmitic acid during the 3 h incubation. Moreover, higher sperm progressive motility and VSL were observed in both OA- and PA-treated groups than those in the control (Figure 8D,E), whereas the total motility did not change (Figure 8F). 

### 3.6. Effects of CPT1 Selective Inhibitor, Etomoxir, on Sperm Cpt1 Activity, Mitochondrial Activity, ATP Level, as well as Motility Patterns

**Figure 9:**
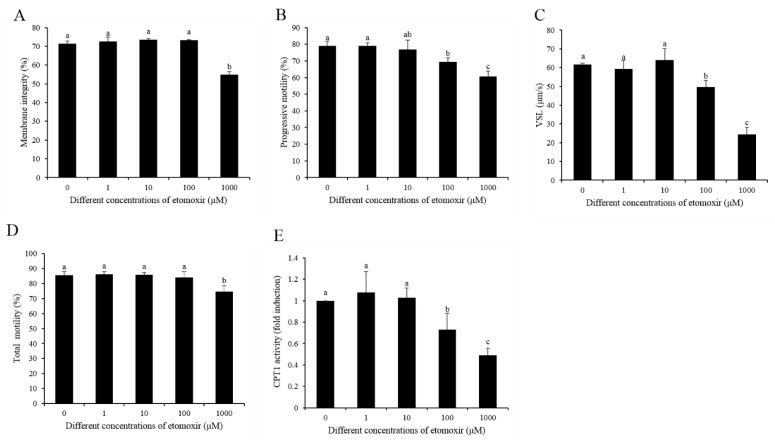
(**A**–**C**) Effects of different concentrations of etomoxir, an inhibitor of β-oxidation, on sperm membrane integrity (**A**), progressive motility (**B**), VSL (**C**), total motility (**D**), and CPT1 activity (**E**). Data are the mean ± standard deviation (SD). Columns with different lowercase letters differ significantly (*p* < 0.05).

**Figure 10:**
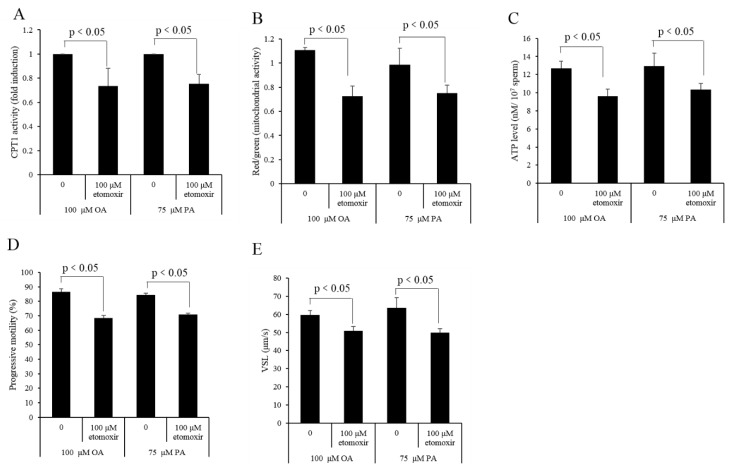
Sperm CPT1 activity (**A**), mitochondrial activity (**B**), ATP level (**C**), progressive motility (**D**), and VSL (**E**) were decreased by the addition of etomoxir to the medium in the presence of OA or PA. Data are the mean ± standard deviation (SD). Columns with different lowercase letters differ significantly (*p* < 0.05).

Etomoxir, an inhibitor of CPT1, was used to re-evaluate the β-oxidation of the oleic acid and palmitic acid that sperm utilized for ATP production. It was observed that the percentage of dead sperm in the 1000 μM etomoxir treatment was the highest, while those in the other treatments were similar to the control (Figure 9A,D), indicating that 1000 μM etomoxir was toxic to sperm. Meanwhile, sperm progressive motility and VSL were decreased in 100 and 1000 μM etomoxir treatments, but the other treatment was similar to the control (Figure 9B,C). Moreover, the CPT1 activity was decreased only in 100 and 1000 μM etomoxir treatments (Figure 9E). Thus, 100 μM etomoxir was used to incubate with sperm in the presence of either 100 μM OA or 75 μM PA subsequently. It was observed that etomoxir significantly decreased the sperm CPT1 activity, mitochondrial activity, and ATP level (Figure 10A–C). Moreover, the addition of etomoxir also decreased the sperm progressive motility and VSL (Figure 10D,E).

## 4. Discussion

**Figure 11:**
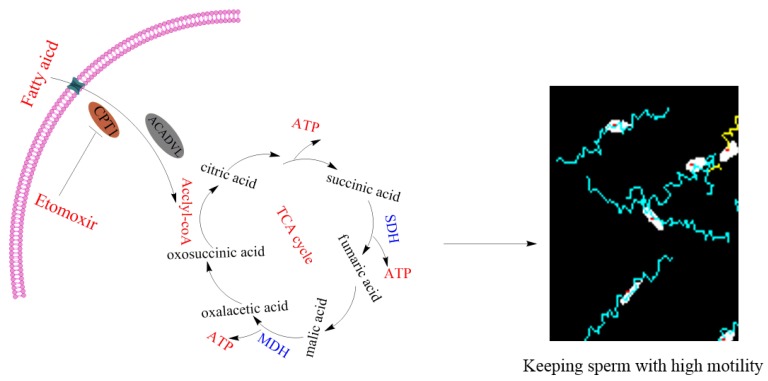
Mechanisms of β-oxidation involved in regulating boar sperm motility. Boar sperm could utilize the fatty acids to generate ATP via β-oxidation, thus maintaining sperm motility. MDH: malate dehydrogenase. SDH: succinate dehydrogenase. ATP: adenosine triphosphate.

In this study, we found that CPT1 and ACADVL, which are involved in mitochondrial β-oxidation, were expressed and localized at the midpiece of boar sperm. During the liquid storage, the addition of oleic acid and palmitic acid significantly increased sperm motility parameters, membrane integrity, and acrosome integrity, while decreasing apoptosis. Moreover, the activity of CPT1 and ACADVL, ATP level, mitochondrial activity, the activity of the TCA cycle enzymes (SDH and MDH), as well as the sperm motility patterns were significantly increased by adding oleic acid and palmitic acid during the 3 h of incubation. Interestingly, the addition of the mitochondrial β-oxidation inhibitor (etomoxir) counteracted the beneficial roles. Based on those data, it was likely that boar sperm might utilize fatty acid for maintaining the movement, and the addition of oleic acid and palmitic acid might improve sperm quality via the mitochondrial β-oxidation metabolic pathway to provide ATP (Figure 11).

The mission for sperm is the delivery of the male genetic information to the offspring [28]. To achieve this, sperm has to swim from the site of ejaculation or insemination towards the oocyte at the fertilization site. Energy metabolism for ATP production is indispensable for sperm migration in the female reproductive tract [29,30]. Glycolysis is generally considered as the primary substrate for ATP production in mammalian sperm [31]. However, we previously reported that the mitochondrial oxidative phosphorylation pathway was also important for boar sperm motility [2]. It has been reported that acetyl-CoA is a metabolite derived from fatty acid catabolism [6] and directly enters the mitochondrial oxidative phosphorylation for ATP production [32] in somatic cells. Moreover, in our previous study [2], we found that boar sperm could switch the energy metabolic pathway from glycolysis to mitochondrial oxidative phosphorylation under the low glucose condition, which suggested that boar sperm has a complex system to use different metabolic substrates (such as fatty acids) for ATP generation under energy stress conditions. Indeed, it was observed that the levels of oleic acid and palmitic acid in boar sperm were decreased after 3, 5, and 7 days of storage in the present study. It might be that sperm utilize them for energy metabolism to produce ATP as boar sperm suffered energy stress during the storage at 17 °C [33].

Interestingly, in the present study, exposure of boar sperm to 100 μM oleic acid or 75 μM palmitic acid resulted in an increase of motility parameters, membrane integrity, and acrosome integrity and a reduction of apoptosis. Those data suggested that boar sperm might consume fatty acids, which was in accordance with human sperm using endogenous components to keep alive for several days in culture medium without any exogenous substrates [34]. Moreover, [1-^14^C] fatty acids could be oxidized in human, ram, bull, dog, and fowl sperm, as indicated by the production of labeled CO_2_ [17]. Therefore, the addition of oleic acid and palmitic acid to the extender might be beneficial for improving sperm quality during the liquid storage.

Fatty acids are metabolized by mitochondrial β-oxidation [35], where CPT1 and ACADVL are the key enzymes [36]. CPT1 is responsible for transporting long-chain fatty acids into the mitochondrial matrix [37], while ACADVL catalyzes fatty acid β-oxidation in the first step [38]. In the present study, we found that CPT1 and ACVAVL were localized in the sperm midpiece where the mitochondria are located, indicating that sperm has the mitochondrial β-oxidation system, which agrees with the findings in human sperm [17,18]. In the present study, when boar sperm was incubated in the solution containing low concentration of glucose, the addition of oleic acid or palmitic acid enhanced the activities of CPT1 and ACADVL, mitochondrial activity, as well as ATP production. Meanwhile, etomoxir, an inhibitor of β-oxidation, attenuated the positive effects of oleic acid and palmitic acid. Taken together, we concluded that boar sperm utilizes oleic acid and palmitic acid for ATP production via mitochondrial β-oxidation.

The artificial insemination (AI) technique is widely applied in the pig industry. However, the technique is still not efficient due to the large sperm dose required to obtain high reproductive performance [39]. Optimization of the extenders and regulation of sperm motility are the strategies for AI efficiency. The novel insight from this study might provide a new strategy for improving the AI technique in livestock animals. 

## 5. Conclusions

Take together, the mitochondrial β-oxidation metabolic pathway works in boar sperm. Sperm could utilization of oleic acid and palmitic acid as the energy substrates for ATP production. Addition of fatty acids to the extender would increase boar sperm quality during the liquid storage.
